# The impact of childhood malnutrition on mortality from pneumonia: a systematic review and network meta-analysis

**DOI:** 10.1136/bmjgh-2021-007411

**Published:** 2021-11-30

**Authors:** Amir Kirolos, Rachel M Blacow, Arun Parajuli, Nicky J Welton, Alisha Khanna, Stephen J Allen, David A McAllister, Harry Campbell, Harish Nair

**Affiliations:** 1 University of Liverpool Department of Women's and Children's Health, Liverpool, UK; 2 Malawi-Liverpool-Wellcome Trust Clinical Research Programme, Blantyre, Malawi; 3 The University of Edinburgh Usher Institute, Edinburgh, UK; 4 University of Bristol Bristol Population Health Science Institute, Bristol, UK; 5 Clinical Sciences, Liverpool School of Tropical Medicine, Liverpool, UK; 6 Alder Hey Children's Hospital, Liverpool, Merseyside, UK; 7 University of Glasgow Institute of Health and Wellbeing, Glasgow, UK

**Keywords:** epidemiology, nutrition, public health, systematic review, pneumonia

## Abstract

**Introduction:**

Childhood malnutrition is widespread in low-income and middle-income countries (LMICs) and increases the frequency and severity of infections such as pneumonia. We aimed to identify studies investigating pneumonia deaths in malnourished children and estimate mortality risk by malnutrition severity.

**Methods:**

We conducted a systematic review of MEDLINE, EMBASE and Global Health databases to identify relevant studies. We used a network meta-analysis to derive ORs of death from pneumonia for moderately and severely underweight children using low weight-for-age, the most reported measure of malnutrition. We compared meta-estimates of studies conducted before and after 2000 to assess changes in mortality risk over time. We estimated the prevalence of underweight hospitalised children from hospital-based cohort studies and calculated the population attributable fraction of in-hospital pneumonia deaths from being underweight using our results.

**Results:**

Our network meta-analysis included 33 544 underweight children from 23 studies. The estimated OR of death from pneumonia was 2.0 (95% CI 1.6 to 2.6) and 4.6 (95% CI 3.7 to 5.9) for children moderately and severely underweight, respectively. The OR of death from pneumonia for those severely underweight was 5.3 (95% CI 3.9 to 7.4) pre-2000 and remained high post-2000 at 4.1 (95% CI 3.0 to 6.0). Prevalence of underweight children hospitalised with pneumonia varied (median 40.2%, range 19.6–66.3) but was high across many LMIC settings. We estimated a median 18.3% (range 10.8–34.6) and 40.9% (range 14.7–69.9) of in-hospital pneumonia deaths were attributable to being moderately and severely underweight, respectively.

**Conclusions:**

The risk of death from childhood pneumonia dramatically increases with malnutrition severity. This risk has remained high in recent years with an estimated over half of in-hospital pneumonia deaths attributable to child malnutrition. Prevention and treatment of all child malnutrition must be prioritised to maintain progress on reducing pneumonia deaths.

**Key questions**

**What is already known?**

- Malnutrition is widespread in low-income and middle-income countries and increases the frequency and severity of infections such as pneumonia.

**What are the new findings?**

- Children who are moderately underweight hospitalised with pneumonia are twice as likely to die, and children who are severely underweight are four and a half times as likely to die compared with children with a normal weight.
- The risk of death has remained high in recent years comparing studies conducted before and after the year 2000. This is despite improved overall care seeking and hospital care for childhood pneumonia over this time.
- An estimated two in five children admitted to hospital with pneumonia in low-income and middle-income countries are moderately or severely underweight.

**What do the new findings imply?**

- Over half of in-hospital child pneumonia deaths in low-income and middle-income countries are attributable to being underweight.
- Prevention and treatment of all child malnutrition must be prioritised to maintain progress on reducing pneumonia deaths.

## Introduction

Pneumonia is a leading cause of child hospitalisations and deaths globally.^1 2^ There were an estimated 808 000 child pneumonia deaths in 2017, mainly in low-income and middle-income countries (LMICs).^3^ Malnutrition increases the frequency and severity of common childhood infections such as pneumonia, delays recovery and increases risk of death.^4^ Childhood malnutrition remains widespread, with an estimated 6.7% of children under 5 globally suffering from wasting and 22.0% stunted.^5^ Pneumonia may present differently in malnourished children with less overt signs of respiratory distress, which results in underdiagnosis and suboptimal management.^6^ Acute infections reciprocally worsen nutritional status through higher catabolism, anorexia and nutrient loss, contributing to a worsening cycle of infections and malnutrition.^7^ While there has been substantial progress in reducing pneumonia morbidity and mortality since the year 2000, there have been smaller improvements in linear growth and reductions in wasting and low birth weight over this time.^2 8^

Previous studies have found that children with both moderate and severe malnutrition have a higher risk of death from pneumonia.^4 9^ Improving child nutrition is, therefore, a key aspect of the global strategy to reduce pneumonia deaths.^10^

Quantifying the deleterious effects of malnutrition and pneumonia can inform ongoing strategies and health programmes aimed at reducing child mortality. We aimed to quantify the risk of death from pneumonia in children with different grades of malnutrition severity. We also aimed to estimate the proportion of child pneumonia deaths attributable to moderate and severe malnutrition.

## Methods

We did a systematic literature review of medical databases MEDLINE, EMBASE and Global Health. Searches included studies from 1 January 1980 to 13 September 2020. Detailed search strategies are included in online supplemental appendix 1.

10.1136/bmjgh-2021-007411.supp1 Supplementary data

### Selection criteria

We included studies reporting original data comparing pneumonia deaths of children under 5 years with and without malnutrition. We included studies which measured weight-for-age (w/a), weight-for-height (w/h) or height-for-age (h/a). We included studies that either reported number of deaths or provided OR or relative risk of death with CIs. We excluded studies with less than 40 study participants and studies that did not describe how malnutrition or pneumonia were defined. We also excluded studies where the diagnosis of pneumonia was based solely on caregiver report. No date or language restrictions were placed on studies.

### Literature search

Titles and abstracts were screened by two independent reviewers (two of AKi, AKh, AP and RMB). Selected full texts were then reviewed independently by two reviewers (two of AKi, AKh, AP, RMB and SJA). Disagreements over inclusion were resolved through mutual discussion. We also screened reference lists of included studies.

We contacted study authors to request additional data for studies that met the inclusion criteria but had missing data, or where data were not stratified by malnutrition severity in the original manuscript. For studies with a risk estimate and sample size but no case numbers (and where we were unable to establish contact with study authors for additional data) we imputed mortality numbers from the CIs.^11^

We assessed study quality using the National Institute of Health and Care Excellence (NICE) quality appraisal checklist.^12^ Study quality was independently scored by two reviewers (two of AKh, AKi, AP and RMB) with scoring disagreements resolved through mutual discussion.

### Case definitions

We classified low weight-for-age as being underweight, low weight-for-height as wasting and low height-for-age as stunting. We considered a grade of moderate for these three measures to be a z score of between −2 and −3 SD, or 60%–80% of expected for the reference population (eg, National Centre for Health Statistics or WHO child growth reference charts). A grade of severe was considered as a z score of less than −3 SD, or less than 60% of expected for the reference population, or the presence of nutritional oedema (for those with general malnutrition or wasting).

Case definitions for pneumonia varied between studies and included definitions are fully documented in online supplemental table 1.

### Data analysis

We used Bayesian hierarchical generalised linear models to estimate the OR of death from pneumonia in children with low weight-for-age. We used low weight-for-age for this analysis as this was the most common anthropometric measure and few studies reported weight-for-height.

Based on this definition for underweight children, most studies provided results for ‘none’, ‘moderate’ and ‘severe’ malnutrition. However, around a third only provided results after collapsing these into two categories (eg, ‘none-moderate’ vs ‘severe’ or ‘none’ vs ‘moderate-severe’). To include all studies we used a Bayesian shared parameter framework^13^ which could accommodate studies reporting results for all three categories and studies reporting results for collapsed categories in a single model.^14–18^ We used a network meta-analysis model^13^ for comparisons defined by underweight categories (rather than treatments) with a binomial likelihood for each study and category and a logit link to estimate the log-ORs for mortality for ‘moderate’ vs ‘none’ (d1) and ‘severe’ vs ‘none’ (d2). A random effects model was fitted to allow log-ORs to vary between study, giving flat Normal priors for the overall mean log-ORs (d1, d2) and a uniform (0,2) prior for the between studies SD. For studies reporting results for collapsed categories, we assumed these estimate a weighted average of the log-ORs for the categories that were combined, weighted by the proportions in each of the combined categories. Because the proportion of individuals in the collapsed categories was not reported, we predicted this based on a model to estimate the proportion in the ‘moderate’ category of each of the ‘moderate-severe’ and ‘non-moderate’ categories (proportions p1 and p2, respectively), informed by the studies which reported results for all three categories. A hierarchical model normal was used to capture variability between studies for p1 and p2 on the log-odds scale, giving flat Normal priors for the overall mean log-odds and a truncated flat Normal prior for the between studies SD. The resulting predictive distribution is used to predict the proportions in the collapsed category studies. In sensitivity analyses, we assumed that p1 and p2 each lay, with equal probability, between 0 and 1 (ie, we placed a uniform prior on each).

We repeated the analysis after stratifying studies into those undertaken before and after the year 2000 (no studies conducted wholly during the year 2000 were identified) and performed separate network meta-analyses for studies before and after this time. We chose the year 2000 as this was a significant milestone around the millennium development goals and allowed enough studies before and after this time to make a comparison. We calculated the difference between ORs of death before and after 2000 for moderate and severe malnutrition. To calculate the CIs for the difference in ORs, we obtained 1000 samples from a (log) normal distribution for the (log) OR for the effect of being underweight on the risk of death for estimates before and after the year 2000 using the BootComb package in R.^19^ The uncertainty limits for the difference in log ORs were then obtained as the 95% highest density interval.

We undertook standard random effects pairwise meta-analyses (‘none’ vs ‘moderate’, ‘none’ vs ‘severe’, ‘none’ vs ‘moderate-severe’ and ‘none-moderate’ vs ‘severe’) as a comparison to the more complex network meta-analysis where all studies were simultaneously modelled.

We identified hospital cohort studies which measured weight-for-age in all children admitted with pneumonia from our selected studies. We derived the prevalence of underweight children in those admitted to hospital with pneumonia and calculated exact binomial 95% CIs.

We calculated a population attributable fraction (PAF) of in-hospital pneumonia deaths for those moderately and/or severely underweight (depending on stratified data available) for each study using the OR and CIs from the network meta-analysis and the prevalence and CIs derived from individual cohort studies. We used the BootComb package in R to propagate uncertainty of the CIs around OR and prevalence estimates.^19^ We obtained 1000 samples from a (log) normal distribution for the (log) OR for the effect of being underweight on the risk of death, and 1000 samples from a beta distribution for the proportion in each malnutrition category. For each sample the PAF was estimated as PAF = [Malnutrition prevalence x (OR-1)]/[Malnutrition prevalence x (OR-1)+1] and uncertainty limits for the PAF were obtained as the 95% highest density interval. The parameters for the log-normal distribution were obtained directly from the network meta-analysis. The parameters for the beta distribution were estimated to match the upper and lower confidence limits from the prevalence analysis described above.

Statistical models and analyses were run in R V.4.0.4 and Openbugs. Modelling is described in detail online with data and code used available at: https://github.com/dmcalli2/malnutrition.

## Results

### Study characteristics

Twenty-six studies met our selection criteria (online supplemental figure 1).^20–45^ All studies were hospital-based and, apart from one case–control study, were prospective or retrospective cohorts (online supplemental table 1). Study recruitment years ranged between 1981 and 2016 and included participants throughout Africa, Asia and South America.

Twenty-three studies provided data on in-hospital mortality by weight-for-age (underweight), six by weight-for-height (wasting) and six by height-for-age (stunting). Most studies relied on a clinical diagnosis of pneumonia, but there was variation of case definitions used in included studies (online supplemental table 1). Six studies used or included confirmation with chest x-ray as part of their diagnostic criteria.

Study quality assessment scoring is included in online supplemental table 2. Most studies had similar strengths and limitations due to similar study designs. Most studies had good external validity with well described study populations and representative study samples. Many studies had mixed internal validity with few studies identifying and adjusting for confounding factors, some studies with low sample sizes but otherwise most with reliable and complete outcome measures.

### Pneumonia mortality risk

**Figure 1:**
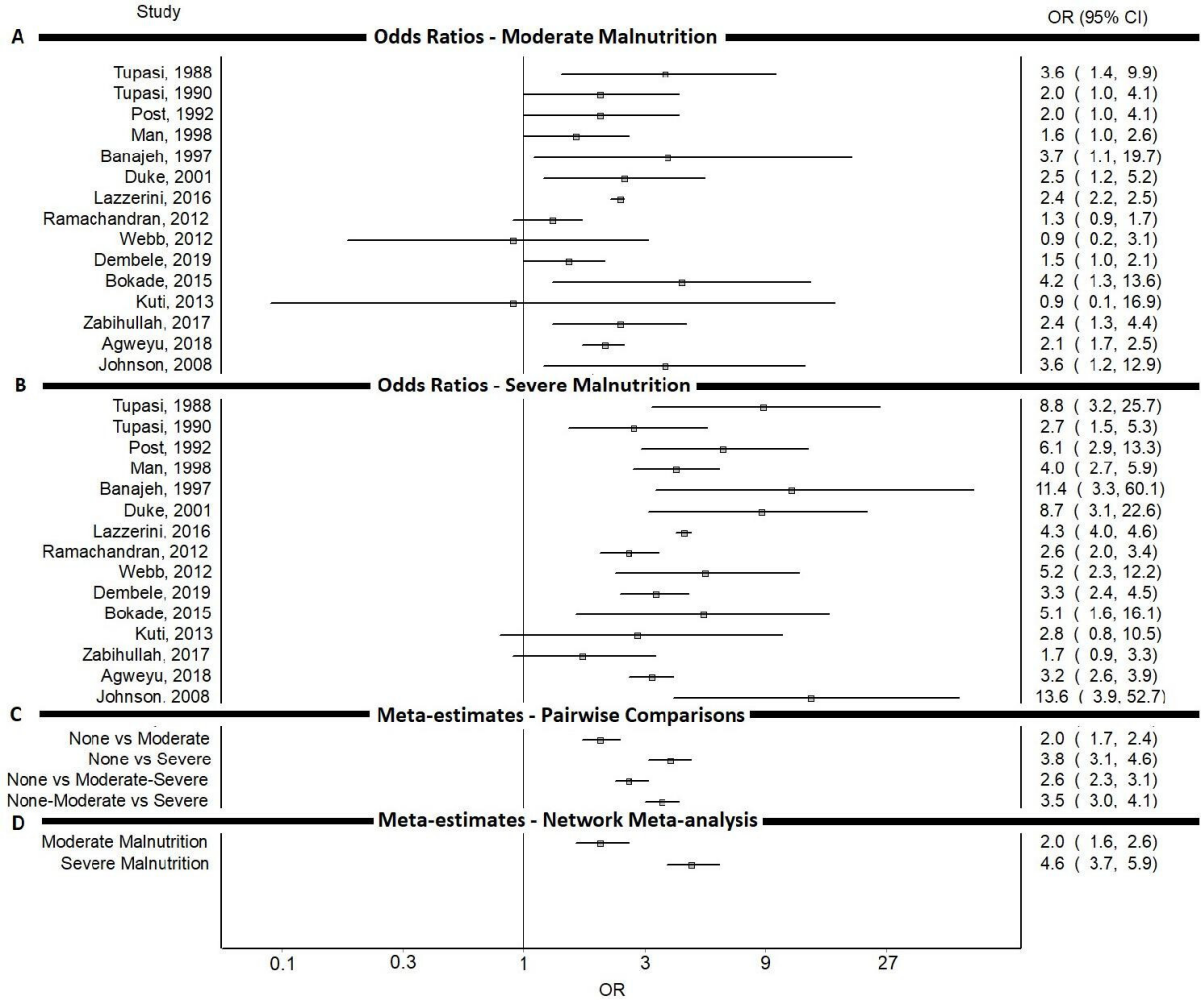
OR of death from pneumonia for (A) studies comparing children moderately underweight (low weight-for-age) to those with normal weight (B) studies comparing children severely underweight to those with normal weight (C) meta-estimates from meta-analyses of studies with pairwise comparisons (D) meta-estimates from network meta-analysis for being moderately and severely underweight. ((A) and (B) only show results from studies which do not collapse moderate and severe underweight categories).

Most studies showed a higher OR (apart from two which showed a lower OR and five in which CIs included 1.0) of death in those moderately underweight compared with controls (figure 1, online supplemental table 3). All studies showed a higher OR of death (apart from two in which CIs included 1.0) for those severely underweight (figure 1, online supplemental table 3). The risk of death was higher for those with severe low weight-for-age compared with those moderately low weight-for-age in all included studies (figure 1, online supplemental table 3).

The network meta-analysis included 33 544 children with low weight-for-age from 23 studies. We estimated an OR of death from pneumonia of 2.0 (95% CI 1.6 to 2.6) for moderately underweight children and 4.6 (95% CI 3.7 to 5.9) for those severely underweight (figure 1). Standard random effects meta-analyses using direct comparisons reported in studies were consistent with the results from the network meta-analysis (figure 1, online supplemental figures 2−5). Sensitivity analyses which placed uniform priors of the proportion of children in collapsed categories did not significantly affect the results of the network meta-analysis reported above (sensitivity analyses are available online at: https://github.com/dmcalli2/malnutrition).

Height-for-age showed an inconsistent relationship with mortality risk (online supplemental table 3, online supplemental figure 6). Five of six studies showed higher OR of death for stunted children, but CIs were wide for these estimates. These risk estimates were also unadjusted for the co-occurrence of wasting which is often present in stunted children.^46^

### Pneumonia mortality risk by year

Based on 10 studies, we estimated an OR of death of 2.1 (95% CI 1.5 to 2.9) for moderately underweight children before the year 2000. This was compared with an estimated OR of 1.9 (95% CI 1.3 to 2.8) based on 12 studies after the year 2000 (One study Johnson *et al*,^31^ did not specify the years over which it was conducted and was therefore not included in the analysis comparing studies before and after the year 2000). We found poor evidence of a significant difference in the OR of death for moderately underweight children before and after 2000 based on a difference of −0.2 (95% CI −1.2 to 0.9).

For those severely underweight, we estimated an OR of death of 5.3 (95%CI 3.9 to 7.4)) before the year 2000 compared with 4.1 (95% CI 3.0 to 6.0) after. We found poor evidence of a significant difference in the OR of death for those severely underweight before and after 2000 based on a difference of −1.2 (95% CI −3.5 to 1.1).

### Prevalence of malnutrition in children admitted with pneumonia

The prevalence of underweight children admitted with pneumonia was generally high but varied considerably between studies and countries (median 40.2%, range 19.6–66.3) (table 1). The median prevalence of those moderately underweight was 22.4% (range 12.1–52.9) and the median prevalence of those severely underweight was 19.3% (range 4.8–64.6) across studies.

**Table 1:** Prevalence of malnutrition (low weight-for-age) and population attributable fraction (PAF) of deaths in children admitted to hospital with pneumonia

Study	Study year	Country	Moderate Malnutrition Prevalence (%) (95% CI)	Moderate malnutrition PAF (%) (95% CI)	Severe malnutrition prevalence (%) (95% CI)	Severe malnutrition PAF (%)(95% CI)	All malnutrition prevalence (%) (95% CI)
Tupasi, 1988^43^	1981–83	Philippines	30.2 (26.9 to 33.7)	23.2 (14.9 to 32.0)	11.4 (9.2 to 13.9)	29.1 (22.1 to 36.7)	41.6 (38 to 45.2)
Collings, 1985^27^	1982–1983	PNG	–	–	–	–	36.9 (32.2 to 41.8)
Tupasi, 1990^42^	1984–86	Philippines	28.2 (24.4 to 32.3)	22 (13.9 to 30.7)	38.1 (33.9 to 42.4)	57.8 (50.3 to 65.4)	66.3 (62.1 to 70.3)
Nathoo, 1993^38^	1989–90	Zimbabwe	–	–	11.6 (9.4 to 14.3)	29.5 (22.6 to 37.3)	–
Agrawal, 1995^20^	1993–95	India	–	–	27.6 (20 to 36.2)	49.8 (39.6 to 60.0)	–
Man, 1998^35^	1993–95	Gambia	23.1 (21.4 to 24.9)	18.8 (11.7 to 26.2)	18.5 (16.8 to 20.1)	39.9 (32.8 to 47.3)	41.6 (39.5 to 43.7)
Lupisan, 2007^34^	1994–00	Philippines	–	–	–	–	29.5 (27 to 32.1)
Banajeh, 1997^23^	1995–96	Yemen	52.9 (48.6 to 57.3)	34.6 (23.9 to 45.4)	22.9 (19.4 to 26.7)	45.2 (37.2 to 53.5)	75.8 (71.9 to 79.4)
Duke, 2001^30^	1998–99	Papua New Guinea	25.0 (21.8 to 28.4)	20 (12.6 to 28.1)	4.8 (3.3 to 6.6)	14.7 (9.4 to 20.7)	29.8 (26.4 to 33.3)
Lazzerini, 2016^33^	2001–12	Malawi	14.1 (13.8 to 14.3)	12.3 (7.5 to 17.7)	8.9 (8.7 to 9.1)	24.2 (19.1 to 29.8)	21.5 (21.2 to 21.8)
Naheed, 2009^36^	2004–07	Bangladesh	–	–	5.0 (4.4 to 5.7)	15.3 (11.4 to 19.7)	–
Chisti, 2010^25^	2005–06	Bangladesh	–	–	64.6 (49.5 to 77.8)	69.9 (61.7 to 77.2)	–
Nantanda, 2008^37^	2005–06	Uganda	–	–	25.5 (18.9 to 33)	47.8 (38.0 to 57.8)	–
Ramachandran, 2012^40^	2006–08	India	26.9 (25.6 to 28.3)	21.2 (13.6 to 29.4)	21.8 (20.6 to 23.2)	44 (36.9 to 51.5)	48.8 (47.2 to 50.3)
Chisti, 2011^26^	2007	Bangladesh	–	–	41.0 (34 to 48.3)	59.6 (51.3 to 67.5)	–
Webb, 2012^44^	2007–08	Kenya	21.7 (18.3 to 25.3)	17.8 (10.8 to 25.3)	19.5 (16.4 to 23)	41.3 (33.5 to 49.6)	41.2 (37.1 to 45.4)
Dembele, 2019^28^	2008–16	Philippines	18.4 (17.3 to 19.5)	15.5 (9.6 to 22.0)	19 (17.9 to 20.1)	40.6 (33.6 to 47.9)	37.4 (36 to 38.7)
Bokade, 2015^24^	2010–12	India	17.6 (13.4 to 22.5)	15 (8.5 to 22.1)	16.9 (12.8 to 21.7)	37.8 (29.0 to 47.2)	34.5 (29 to 40.3)
Kuti, 2013^32^	2010–11	Gambia	20.6 (16.7 to 24.9)	17.1 (10.2 to 24.6)	23.9 (19.8 to 28.5)	46.3 (37.9 to 54.8)	44.5 (39.5 to 49.6)
Zabihullah, 2017^45^	2012–13	Afghanistan	19.4 (16.3 to 22.7)	16.2 (9.8 to 23.3)	19.7 (16.6 to 23.1)	41.5 (33.6 to 49.7)	39.1 (35.2 to 43.1)
Agweyu, 2018^21^	2014–16	Kenya	12.1 (11.6 to 12.6)	10.8 (6.5 to 15.6)	7.5 (7.0 to 7.9)	21.2 (16.4 to 26.3)	19.6 (18.9 to 20.2)
Johnson, 2008^31^	–	Nigeria	44.9 (39.3 to 50.5)	31 (20.6 to 41.3)	11.2 (8.0 to 15.2)	28.8 (20.4 to 38.0)	56.1 (50.5 to 61.6)

### PAF of pneumonia deaths due to child malnutrition

Estimated in-hospital pneumonia deaths attributable to being moderately and severely underweight were high across studies (table 1). We estimated a median 18.3% (range 10.8–34.6) of child pneumonia deaths were attributable to being moderately underweight and a median of 40.9% (range 14.7–69.9) were attributable to being severely underweight.

## Discussion

Children with malnutrition have a substantially higher risk of death from pneumonia which increases with malnutrition severity. The risk of death has remained high in recent years comparing studies conducted before and after the year 2000. Our study estimates of pneumonia mortality risk for both moderately and severely underweight children can be used to measure trends in pneumonia deaths attributable to malnutrition. Given the high prevalence of all severities of underweight children hospitalised with pneumonia across LMIC settings, and the disproportionately high number of deaths attributable to being severely underweight, there are significant implications for pneumonia programmes in terms of policies prioritising the prevention and treatment of all forms of malnutrition.

Studies identified recruited participants from hospitals and so our results are only reflective of in-hospital mortality risk. Based on an estimate of 265 000 in-hospital pneumonia deaths in 2010,^1^ and using our median PAF estimates of 18.3% for those moderately underweight and 40.9% for those severely underweight, we estimate 48 495 and 108 385 of in-hospital pneumonia deaths were attributable to being moderately and severely underweight, respectively, in 2010. These estimates do not include community deaths and do not account for the risk of postdischarge mortality which is higher for children with severe malnutrition treated for pneumonia.^47^ Our estimates for the OR of death may be different to the OR of death in the community and future community-based studies can investigate to what extent these may differ. Studies can also look at incidence rates of pneumonia in children with malnutrition and the effect of nutritional therapy in mitigating these risks in the community. One limitation of our meta-estimates is that risk is not stratified by HIV status, a risk factor strongly associated with both malnutrition and risk of death from pneumonia. Very few studies we identified stratified results by or adjusted for HIV status. Future studies should investigate the difference in risk for malnourished children living with and without HIV as pneumonia mortality risk will likely differ between settings depending on HIV prevalence.^48^

Our reported hospitalised malnutrition prevalence estimates, and therefore, our PAF estimates are based on numbers reported from included hospital cohort studies. These studies may have biases in terms of case recruitment which may underestimate or overestimate in-hospital underweight prevalence as these were not the primary aims of studies. Results showed a particularly high attributable fraction of deaths for severely underweight children, and given the much higher prevalence of moderate malnutrition compared with severe, bias in case recruitment for studies may have increased the number of deaths we attribute to being severely underweight. Our results can be refined in future by applying our reported OR of death to specific country prevalence estimates of malnutrition which have been systematically assessed. Few studies used weight-for-height and two study authors we contacted reported likely biases and difficulties in measuring height accurately for acutely unwell children in these studies (Berkley, Dhoubhadel, Personal communication). We therefore used weight-for-age in our network meta-analysis to compare results across the largest number of studies. There were differences in the reference populations (eg, WHO vs NCHS standards) and pneumonia case definitions between studies, but these are unlikely to significantly affect interpretation of our results.

Severe stunting was associated with a slightly higher risk of death in five of six studies. However, the likely co-occurrence of wasting and other confounders is not accounted for in these studies.^46^ Stunting may be indicative of previous episodes of wasting or milder forms of malnutrition over a longer time. Children with both stunting and wasting have been found to have a higher risk of death than those with severe wasting alone.^49^. It is, therefore, unclear whether stunting is independently associated with risk of death from pneumonia and further studies are required to investigate this. These would also require a larger number of participants given the lesser effect compared with more acute forms of malnutrition.

Our methodology has not been used before to estimate mortality risk associated with different malnutrition severities. Our estimates are based on studies spanning several years with data from a large number of participants. Given that studies stratified results differently or incompletely, the use of a Bayesian model allowed data from all studies to be incorporated and the inclusion of additional stratified data provided by several study authors is likely to have improved our modelled estimates.

Incidence and mortality of childhood pneumonia has decreased in recent years.^2^ Hospitalisation rates have meanwhile increased over the same period and in-hospital case fatality rates have significantly decreased, particularly in low-income countries.^2^ These trends in childhood pneumonia are likely due to earlier presentation to hospitals and improved quality of care. Despite this progress, our results found poor evidence of a significant change in the risk of death in malnourished children in recent years. Our analysis by year of study is limited by the number of data points identified through this review and it is unclear whether there has been a difference in risk in recent years. Interpretating a change in OR over this time may also be affected by differing admission criteria based on malnutrition severity and differences in community-based versus hospital-based treatment, but our results still indicate that there is an ongoing high risk of death based on studies from recent years.

Based on our estimates, over half of all in-hospital pneumonia deaths are attributable to child malnutrition, with at least one-quarter of in-hospital deaths attributable based on lower confidence estimates. Food insecurity and poor sanitation/hygiene in many LMICs is likely to continue affecting child nutrition which may perpetuate high numbers of pneumonia deaths.^50^ Despite better hospital care for pneumonia the risk of death in malnourished children remains high. Prevention and treatment of child malnutrition must therefore be prioritised to maintain progress on reducing pneumonia deaths globally. Mortality risk estimates from this study can be used in future to quantify deaths attributable to malnutrition and to measure the impact of interventions.

## Data Availability

All data relevant to the study are included in the article or uploaded as online supplemental information.
