# Supplementary material for: The impact of childhood malnutrition on mortality from pneumonia: a systematic review and network meta-analysis
Source: BMJ Glob Health. 2021 Nov 30;6(11):e007411. doi: 10.1136/bmjgh-2021-007411 (PMC8634228; doi:10.1136/bmjgh-2021-007411)
Supplement: Supplementary data [file bmjgh-2021-007411supp001.pdf]

Supplementary Figures

Supplementary Figure 1. Flow Chart of Literature Search

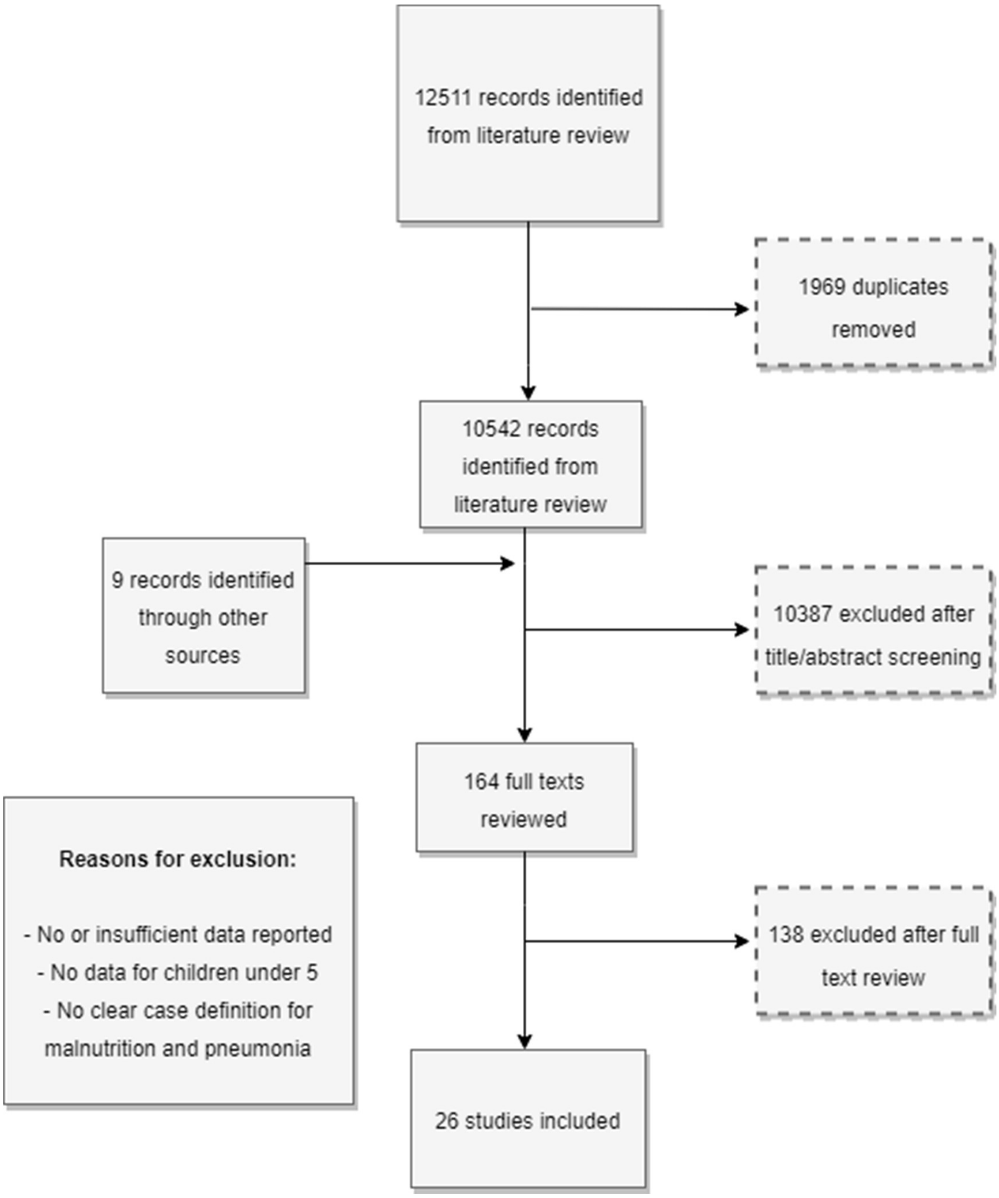

Supplementary Figure 2. Random-effects meta-analysis of studies comparing odds ratio of death from pneumonia for children with moderate malnutrition to those with no malnutrition

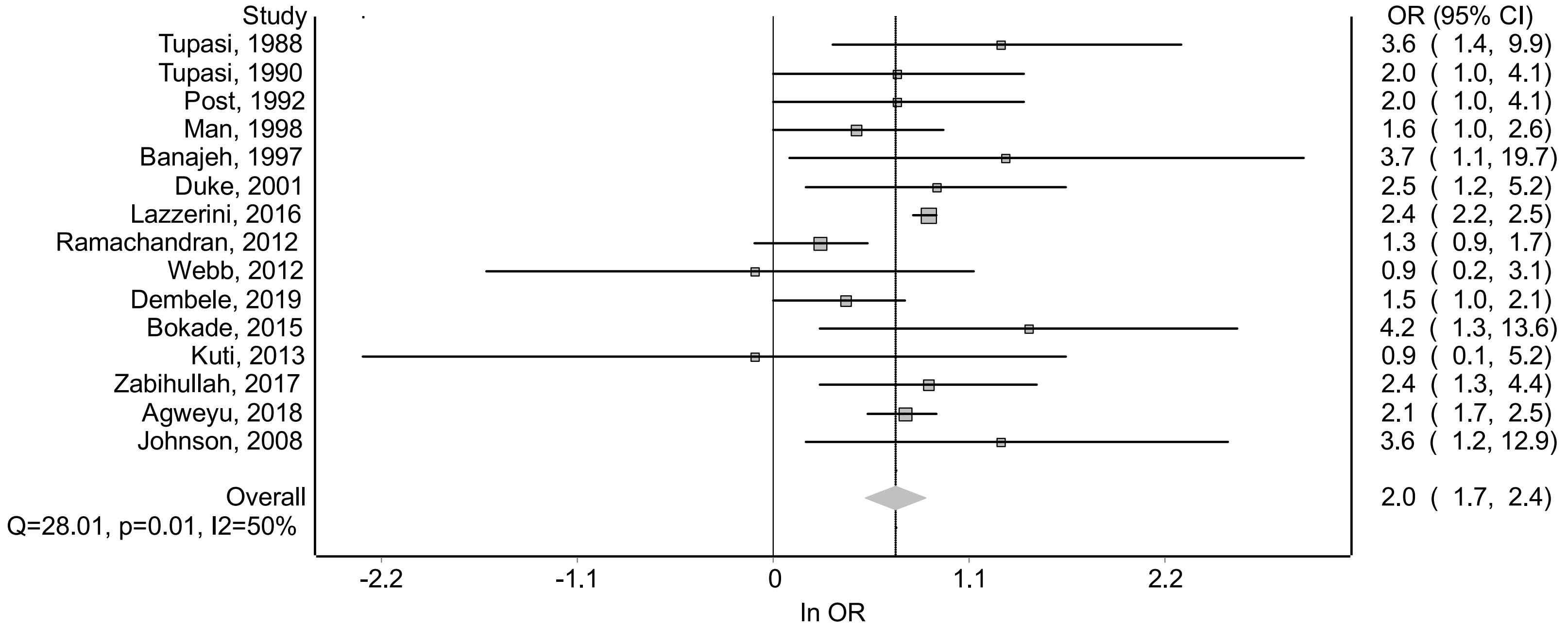

Supplementary Figure 3. Random-effects meta-analysis of studies comparing odds ratio of death from pneumonia for children with severe malnutrition to those with no malnutrition

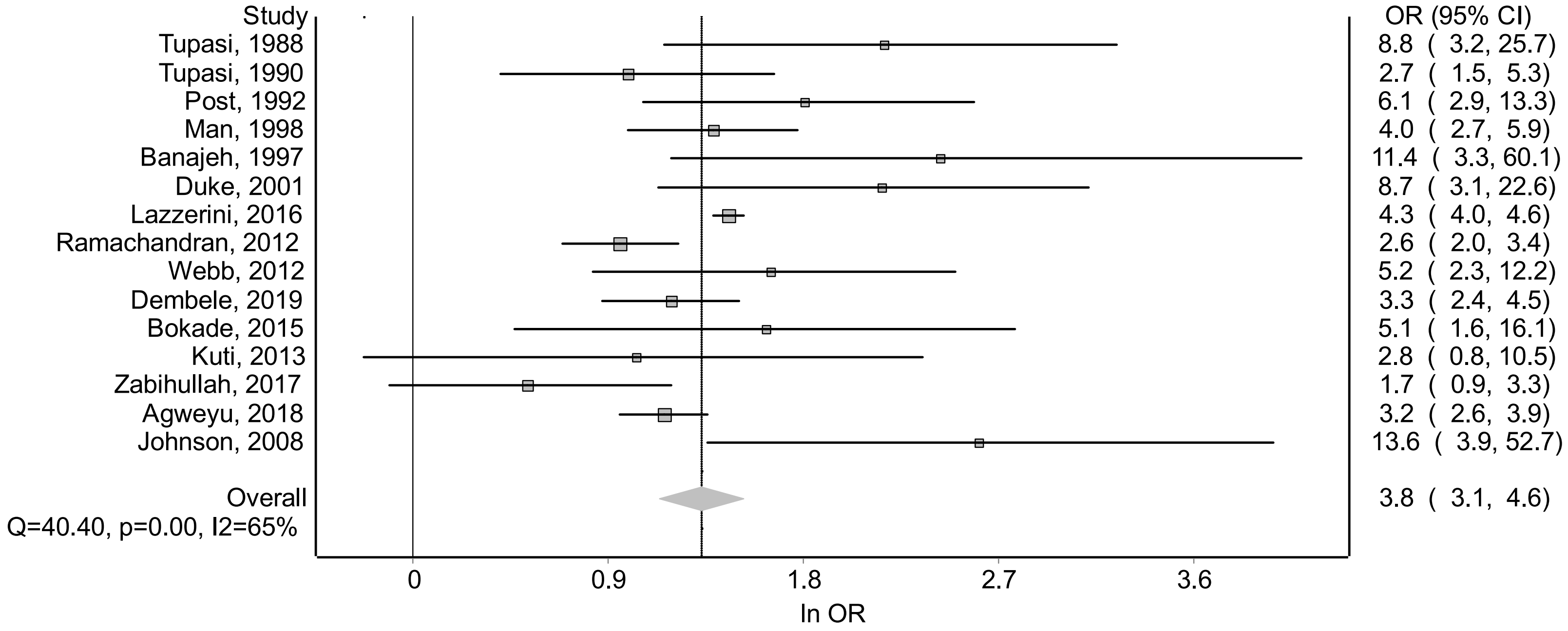

Supplementary Figure 4. Random-effects meta-analysis of studies comparing odds ratio of death from pneumonia for children with any severity of malnutrition to those with no malnutrition

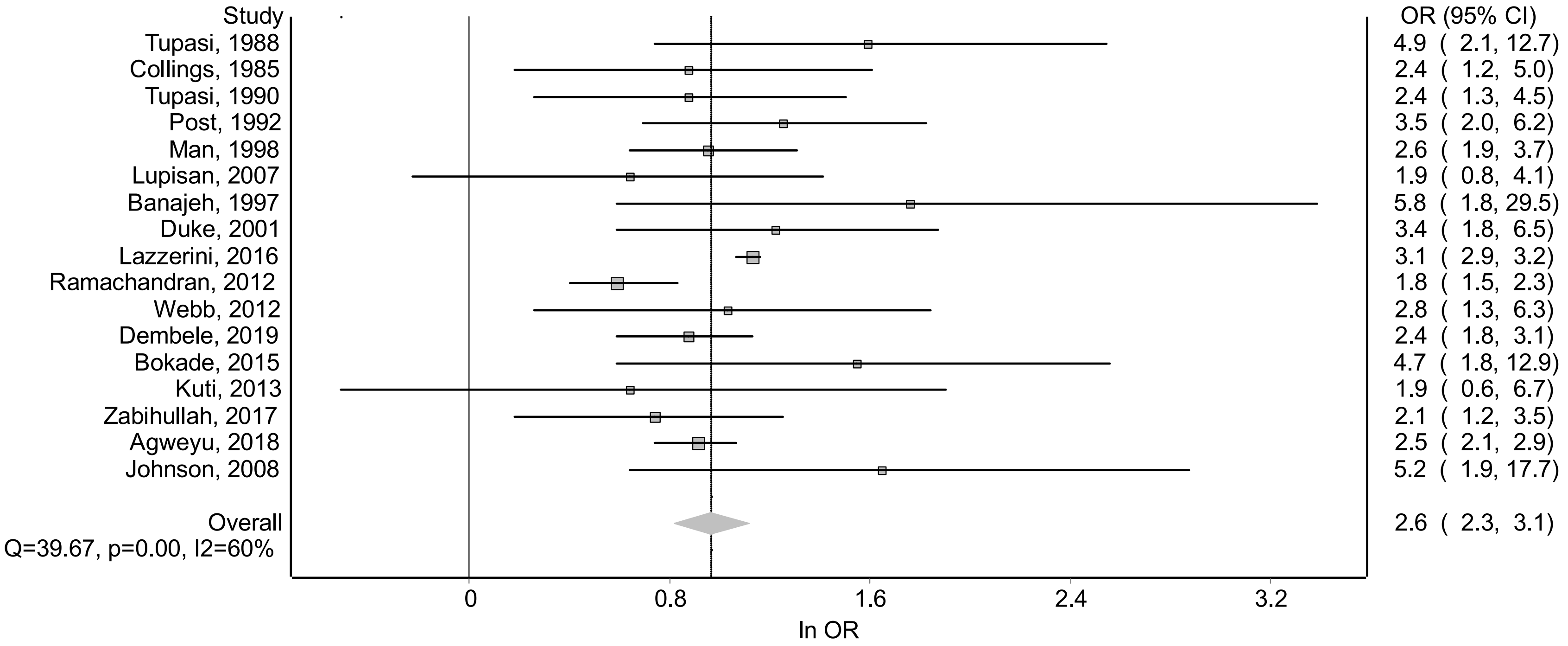

Supplementary Figure 5. Random-effects meta-analysis of studies comparing odds ratio of death from pneumonia for children with severe malnutrition to those without severe malnutrition

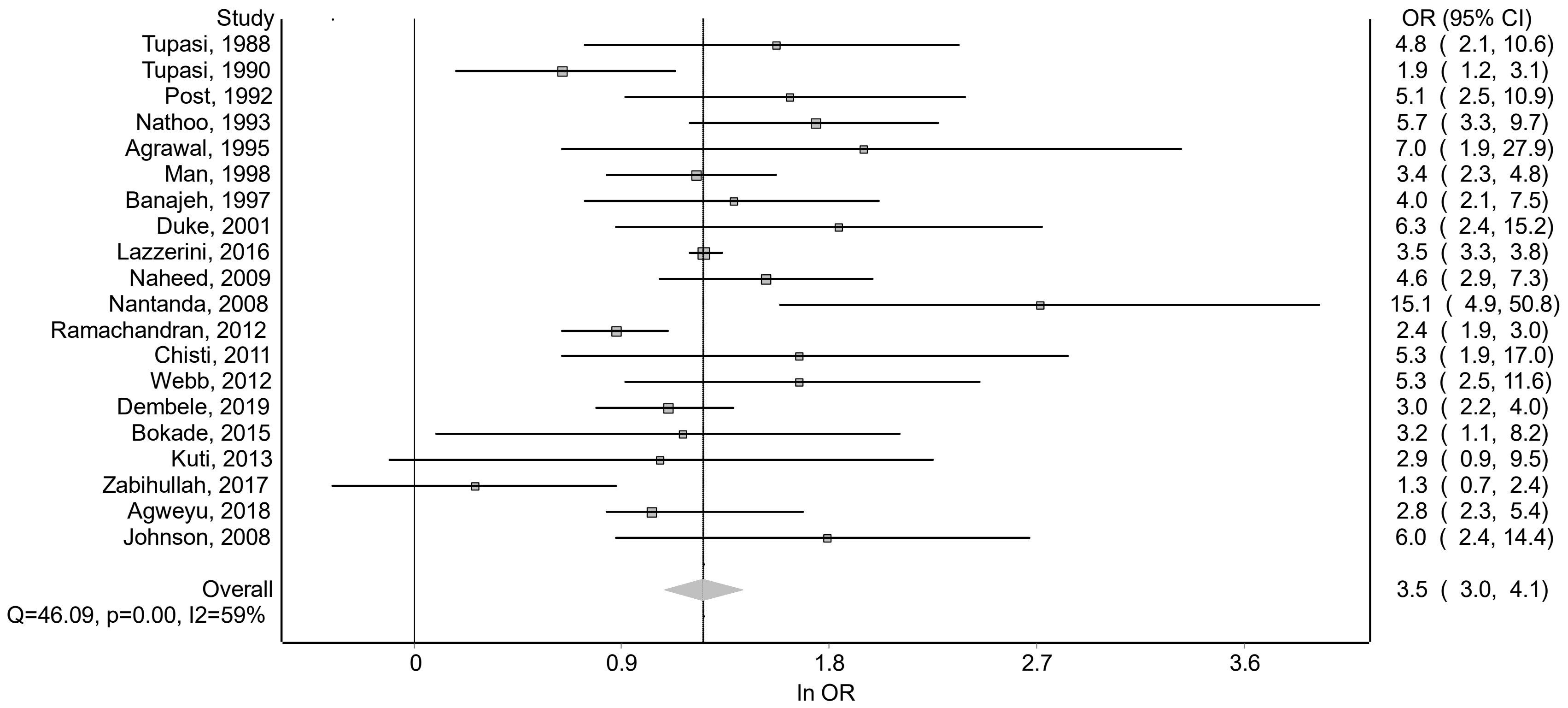

Supplementary Figure 6. Studies comparing odds ratio of death from pneumonia for children with severe stunting compared to those children without stunting

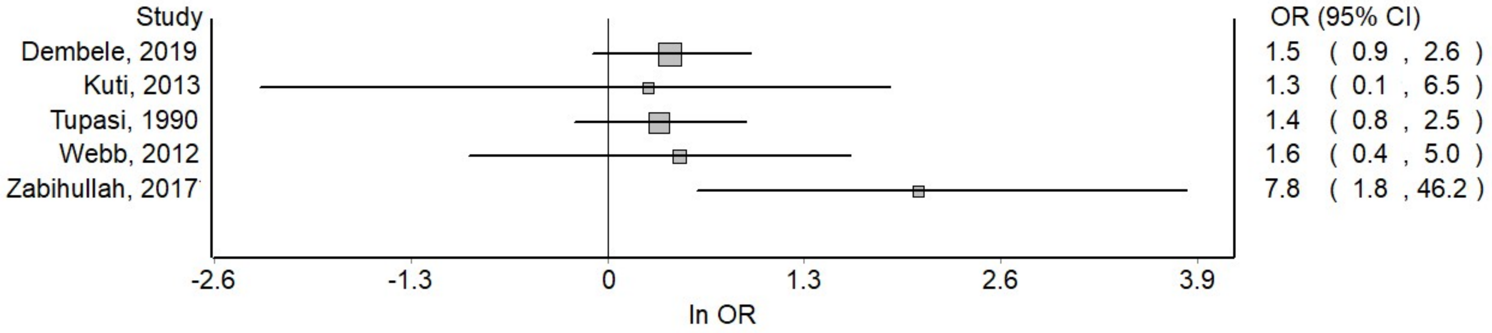

* Figure excludes Demers *et al* (2000) which collapses moderate and severe stunting

Supplementary Tables

Supplementary Table 1. Study Characteristics

Study	Country	Study Period	Age Range	Study Design	Malnutrition Definition (reference population)	Pneumonia Definition
Agrawal, 1995 [20]	India	Feb 93 - Oct 93	2m-59m	Prospective Cohort	IAP classification, w/a < 60% of expected (grade 3/4), w/a >60% of expected (grade 1/2)<br>(IAP classification; based on Harvard growth charts)	WHO Criteria - RR>50 2-12m, RR> 40 13-60m with chest indrawing
Agweyu, 2018 [21]	Kenya	Mar 14 – Feb 16	2m-59m	Retrospective cohort	w/a z score <-2 SD, <-3 SD<br>(WHO 2016)	Lower chest wall indrawing or fast breathing (RR ≥50 breaths and without signs of severe pneumonia per min if aged 2–11 months; RR ≥40 if aged 12–59 months).<br>Severe pneumonia: Any one of: oxygen saturation <90%, central cyanosis, severe respiratory distress, inability to drink or breastfeed or vomiting everything, altered consciousness, and convulsions.
Bahwere, 2004 [22]	Congo	Jan 87 - Dec 97	< 60m	Prospective Cohort	w/h z score <-2 SD; z score <-3 SD (or oedema)<br>(WHO 1999 (NCHS))	Respiratory distress (Tachypnoea, indrawing without anaemia and/or suggestive signs on auscultation)

Banajeh, 1997 [23]	Yemen	Apr 95 - Mar 96	2w-59m	Prospective Cohort	w/a 60-80% of expected, w/a <60% of expected (WHO 1978 (NCHS))	WHO clinical criteria for severe pneumonia
Bokade, 2015 [24]	India	2010-12	1m -59m	Prospective Cohort	w/a z score <-2 SD, <-3 SD (not stated)	WHO Clinical Criteria Severe Pneumonia - age specific tachypnoea with indrawing
Chisti, 2010 [25]	Bangladesh	May 05 - Apr 06	< 12m	Retrospective Cohort	w/a <60% of expected or w/a 60-80% with evidence of oedema (not stated)	Presence of lobar or patchy consolidation on chest radiograph
Chisti, 2011 [26]	Bangladesh	Sep 07 - Dec 07	< 60m	Prospective Cohort	w/a z score < -3 SD (NCHS)	WHO algorithm of acute respiratory infection confirmed on chest radiograph
Collings, 1985 [27]	Papau New Guinea	1982 - 83	1m - 36m	Retrospective Cohort	w/a < 80% of expected (NCHS)	Fever, signs of respiratory distress, clinical chest findings
Dembele, 2019 [28]	Philippines	2008-16	8d – 59m	Cohort	w/a z score <-2 SD, <-3 SD h/a z score <-2 SD, <-3 SD	WHO clinical criteria for severe or very severe pneumonia
Demers, 2000 [29]	CAR	Jul 96 - Jun 97	< 60m	Prospective Cohort	w/h z score <-2 SD h/a z score <-2 SD (not stated)	WHO clinical pneumonia definition and confirmed on x-ray

Duke, 2001 [30]	Papau New Guinea	Jun 98 - Sep 99	28d - 59m	Prospective Cohort	w/a <80% of expected, <60% of expected<br>(not stated)	Cough or difficulty breathing with moderate/severe indrawing plus one of cyanosis, inability to feed, apnoea or signs of heart failure
Johnson, 2008 [31]	Nigeria	30 months (unspecified)	2w - 59m	Prospective Cohort	w/a 60-80% of expected, <60% of expected (or oedema)<br>(Wellcome classification, Harvard Growth Charts)	Pneumonia defined as rales or evidence of consolidation on physical examination or radiograph
Kuti, 2013 [32]	Gambia	Nov 10 - Apr 11	2m - 59m	Prospective Cohort	w/a < -2 SD, h/a < -2 SD, w/h <-3 SD<br>(NCHS – WHO)<br>*(supplementary study data provided by author calculated against WHO 2006 reference population)*	Difficult, rapid breathing with RR >50 for 2m – 11m, RR>40 for 12m - 59m (depending on age using WHO ARI counter) together with at least one of cyanosis, indrawing, inability to feed, and altered consciousness
Lazzerini, 2016 [33]	Malawi	2001-12	< 60m	Retrospective Cohort	w/a <-2 SD, <-3 SD<br>(WHO 2006)	Cough or difficulty breathing with fast breathing for age

Lupisan, 2007 [34]	Philippines	Apr 94 - May 00	2m – 59m	Prospective Cohort	w/a < -2 SD (NCHS)	WHO defined severe or very severe pneumonia
Man, 1998 [35]	The Gambia	1993 - 95	< 60m	Retrospective Cohort	w/a z score < -2 SD, <-3 SD (Wellcome classification; NCHS)	Clinician diagnosed pneumonia
Naheed, 2009 [36]	Bangladesh	May 04 - Apr 07	2m – 59m	Prospective Cohort	w/a z score < -3 SD (WHO)	History of cough or difficulty breathing and tachypnoea (RR>50 for 2-12 months, RR> 40 for 12 - 59 months) or pneumonia danger sign
Nantanda, 2008 [37]	Uganda	Dec 05 – Mar 06	2m-59m	Prospective Cohort	w/a z score < -3 SD** (not stated)	WHO Severe Pneumonia Criteria - Cough or difficult breathing, tachypnoea and chest in-drawing. Very severe – cyanosis and/or inability to feed in addition.
Nathoo, 1993 [38]	Zimbabwe	Mar 89 - Feb 90	1m - 59m	Prospective Cohort	w/a < 60% of expected (NCHS)	WHO clinical definition – cough associated with difficulty breathing. Moderate – RR>50 Severe – Chest indrawing and/or inability to drink
Post, 1992 [39]	Brazil	May 86 – April 87	1m – 59m	Case-Control	w/a z score < -2 SD, <-3 SD (NCHS)	Recorded by ICD code (ICD 9-480 to 9-4S7)
Ramachandran, 2012 [40]	India	Jan 06 - Dec 08	1m-59m	Retrospective Cohort	w/a z score < -2 SD, <-3 SD (WHO 2006)	WHO clinical criteria (RR>=60 in <2 months, >=50 in 2-11 months, >=40 in 12-59 months or severe pneumonia with chest retraction) or radiologically diagnosed pneumonia (based on chest infiltrates by qualified radiologist and clinician)

Sigauque, 2009 [41]	Mozambique	Mar 04 - Mar 06	< 24m	Prospective Cohort	w/h z score < -3 SD<br>(NCHS)	WHO clinical criteria for severe pneumonia - Cough or difficulty in breathing with increased respiratory rate according to age plus chest wall indrawing. Confirmed on x-ray by two primary readers and external WHO radiologist.
Tupasi, 1990 [42]	Philippines	Nov 84 - Oct 86	< 60m	Prospective Cohort	w/a z score <-2 SD, <-3 SD<br>h/a z score <- 2 SD<br>(NCHS)	ALRI WHO algorithm – Cough and breathing difficulty manifesting as tachypnoea (RR >50)<br>Moderate/severe - chest indrawing or cyanosis
Tupasi, 1988 [43]	Philippines	Mar 81 - Feb 83	< 60m	Prospective Cohort	w/a 75-89%, 60-74%, <60% of expected<br>(Filipino data)	WHO definition of clinical pneumonia – Cough or difficulty in breathing in the presence of increased respiratory rate according to age<br>Severe – presence of chest wall indrawing
Webb, 2012 [44]	Kenya	May 07 - May 08	2m - 59m	Prospective Cohort	w/a z score <-2 SD, <-3 SD<br>w/h z score <-2 SD, <-3 SD<br>h/a z score <-2 SD, <-3 SD<br>(WHO)	History of cough or difficulty breathing and lower chest wall indrawing.<br>Very severe – inability to drink, reduced consciousness or central cyanosis

Zabihullah 2017 [45]	Afghanistan	Dec 12 – Mar 13	< 60m	Prospective Cohort	w/a z score <-2 SD, <-3 SD<br>w/h z score <-2 SD, <-3 SD<br>h/a z score <-2 SD, <-3 SD<br>(WHO 2006)	WHO criteria for clinical pneumonia

RR – respiratory rate, w/a – weight-for-age, w/h – weight-for-height, h/a – height-for age, SD – standard deviation, 'm' – months, 'w' – weeks, NCHS – National Centre for Health Statistics child growth reference chart, WHO – World Health Organization child growth reference chart

** definition confirmed with study author

Supplementary Table 2. Quality assessment of included studies

	Population			Method of Selection					Outcomes					Results				Internal Validity	External Validity
**Study**	**1.1**	**1.2**	**1.3**	**2.1**	**2.2**	**2.3**	**2.4**	**2.5**	**3.1**	**3.2**	**3.3**	**3.4**	**3.5**	**4.1**	**4.2**	**4.3**	**4.4**	**5.1**	**5.2**
Agrawal, 1995 [20]	-	++	++	+	++	++	-	++	++	++	++	na	na	+	+	+	++	Mixed	Mixed
Agweyu, 2018 [21]	++	++	++	++	++	++	++	++	++	++	++	na	na	++	++	++	++	Good	Good
Bahwere, 2004 [22]	++	++	++	++	++	++	-	++	++	+	++	na	na	++	+	+	++	Mixed	Good
Banajeh, 1997 [23]	++	++	++	++	++	++	-	++	++	+	++	na	na	+	-	+	++	Mixed	Good
Bokade, 2015 [24]	-	+	+	++	++	++	+	++	++	++	++	na	na	++	+	+	++	Mixed	Mixed
Chisti, 2010 [25]	+	++	++	+	++	++	+	++	+	++	++	na	na	+	+	+	+	Mixed	Good
Chisti, 2011 [26]	++	++	++	++	++	++	++	++	++	++	++	na	na	++	+	+	++	Mixed	Good
Collings, 1985 [27]	+	++	++	+	+	++	-	++	+	+	++	na	na	++	-	+	+	Mixed	Good
Dembele, 2019 [28]	++	++	++	+	++	++	++	++	++	++	++	na	na	++	++	++	++	Good	Good
Demers, 2000 [29]	++	++	++	+	++	++	++	++	++	+	++	na	na	+	+	+	+	Mixed	Good
Duke, 2001 [30]	+	++	++	+	++	++	-	++	++	++	++	+	++	+	+	+	++	Mixed	Good
Johnson, 2008 [31]	++	++	++	+	++	++	+	++	++	++	++	na	na	++	+	+	++	Mixed	Good
Kuti, 2013 [32]	++	++	++	++	++	++	+	++	++	++	++	na	++	+	++	+	++	Mixed	Good
Lazzerini, 2016 [33]	++	++	++	++	++	++	++	++	++	+	++	na	na	++	++	++	++	Good	Good
Lupisan, 2007 [34]	++	++	++	++	++	++	+	++	++	++	++	na	na	++	++	++	++	Good	Good
Man, 1998 [35]	++	++	++	+	++	++	-	++	++	+	++	na	na	++	-	-	++	Mixed	Good
Naheed, 2009 [36]	+	++	++	+	++	++	-	++	++	++	++	na	na	++	-	+	++	Mixed	Good
Nantanda, 2008 [37]	++	++	++	+	++	++	-	++	++	++	++	na	na	+	-	+	+	Mixed	Good
Nathoo, 1993 [38]	+	++	++	+	++	++	-	++	++	++	++	na	na	++	-	+	++	Mixed	Good
Post, 1992 [39]	++	++	++	+	++	++	-	++	++	++	++	na	na	+	-	+	+	Mixed	Good
Ramachandran, 2012 [40]	++	++	++	+	++	++	++	++	++	++	++	na	na	++	++	++	++	Good	Good
Sigauque, 2009 [41]	++	++	++	++	++	++	-	++	++	++	++	na	na	++	-	+	++	Mixed	Good

Tupasi, 1990 [42]	++	++	++	+	++	++	+	++	++	++	++	na	na	+	++	+	++	Mixed	Good
Tupasi, 1988 [43]	++	++	++	++	++	++	+	++	++	++	++	na	na	++	+	+	++	Mixed	Good
Webb, 2012 [44]	++	++	++	++	++	++	++	++	++	++	++	++	na	+	++	++	++	Good	Good
Zabihullah 2017 [45]	++	++	++	++	++	++	++	++	++	++	++	na	na	++	++	++	++	Good	Good

++ = Good, + = Mixed, - = Poor, na = non-applicable

* template used for quality assessment of studies

Quality Appraisal of Correlation Studies

++ = good, + = mixed, - = poor, nr = not reported, na = not applicable

Cells are colour-coded to demonstrate the relationship with the summary questions below.

	**Study identification** *(include full citation details)*		
	**Study design:**		
	**Evaluation criteria**	**Quality ++ + - nr na**	**Guidance topic:** / **Assessed by:**
Population	**Section 1: Population**		
	**1.1** Is the source population or source area well described?		
	**1.2** Is the eligible population or area representative of the source population or area?		
	**1.3** Do the selected participants or areas represent the eligible population or area?		
Exposure (& Comparison)	**Section 2: Method of selection of exposure (or comparison) group**		
	**2.1** Selection of exposure (and comparison) group. How was selection bias minimised?		
	**2.2** Was the selection of explanatory variables based on sound theoretical basis?		
	**2.3** Was the contamination acceptably low?		
	**2.4** How well were likely confounding factors identified and controlled?		
	**2.5** Is the setting applicable to country in general?		
Outcomes	**Section 3: Outcomes**		
	**3.1** Were the outcome measures and procedures reliable?		
	**3.2** Were the outcome measurement complete?		
	**3.3** Were all important outcomes assessed?		
Time	**3.4** Was there a similar follow-up time in exposure & comparison groups?		
	**3.5** Was follow-up time meaningful?		
Results	**Section 4: Analyses**		
	**4.1** Was the study sufficiently powered to detect an effect if one exists?		
	**4.2** Were multiple explanatory variables considered in the anlayses?		
	**4.3** Were the analytical methods appropriate?		
	**4.4** Was the precision of association given or calculable? Is association meaningful?		
**Summary**	**Section 5: Summary**		
	**5.1 Are the study results internally valid (i.e unbiased)?**		
	**5.2 Are the results generalisable to the source population (i.e externally valid)?**		

Supplementary Table 3. Mortality risk of pneumonia in malnourished children

Study	Measure	Control group	Malnourished group	Malnutrition Category	Unadjusted OR	95% CI	Control Number Survived	Control Number Died	Malnourished Number Survived	Malnourished Number Died	Total number
Agrawal, 1995 [20]	% expected	>60%	<60%	w/a	**7**	1.9 - 27.9	87	5	25	10	127
Agweyu, 2018 [21]	z-score SD	>-2	<-2 to <-3	w/a	**2.1**	1.7 - 2.5	11766	469	1703	140	14078
		>-2	<-2	w/a	**2.5**	2.1 - 2.9	11766	469	2710	267	15212
		>-2	<-3	w/a	**3.2**	2.6 - 3.9	11766	469	1007	127	13369
		>-3	<-3	w/a	**2.8**	2.3 - 3.4	13469	609	1007	127	15212
Bahwere, 2004 [22]	z-score SD	>-2	<-2 to <-3 (no oedema)	w/h	**1.2**	0.5- 2.6	477	43	89	10	619
		>-2	<-2 (or oedema)	w/h	**2.6**	1.7 - 4.1	477	43	221	52	793
		>-2	<-3 (or oedema)	w/h	**3.5**	2.1 - 5.8	477	43	132	42	694
		>-3	<-3 (or oedema)	w/h	**3.4**	2.1 - 5.4	566	53	132	42	793
Banajeh, 1997 [23]	% expected	>80%	60-80%	w/a	**3.7**	1.1 - 19.7	125	3	257	23	408
		>80%	<80%	w/a	**5.8**	1.8 - 29.5	125	3	352	49	529
		>80%	<60%	w/a	**11.4**	3.3 - 60.1	125	3	95	26	249
		>60%	<60%	w/a	**4**	2.1 - 7.5	382	26	95	26	529

Study	Measure	Control group	Malnourished group	Malnutrition Category	Unadjusted OR	95% CI	Control Number Survived	Control Number Died	Malnourished Number Survived	Malnourished Number Died	Total number
Bokade, 2015 [24]	SD/ z-score	>-2	<-2 to <-3	w/a	**4.2**	1.3 - 13.6	182	8	43	8	241
		>-2	<-2	w/a	**4.7**	1.8 - 12.9	182	8	83	17	290
		>-2	<-3	w/a	**5.1**	1.6 - 16.1	182	8	40	9	239
		>-3	<-3	w/a	**3.2**	1.1 - 8.2	225	16	40	9	290
Chisti, 2010 [25]	% expected	>60%	<60%	w/a	**-**	-	17	0	24	7	48
Chisti, 2011 [26]	SD/ z-score	>-3	<-3	w/a	**5.3**	1.9 - 17	109	6	62	18	195
Collings, 1985 [27]	% expected	>80%	<80%	w/a	**2.4**	1.2 - 5	239	17	128	22	406
Dembele, 2019 [28]	z-score SD	>-2	<-2 to <-3	w/a	**1.5**	1 - 2.1	2935	97	850	41	3923
		>-2	<-2	w/a	**2.4**	1.8 - 3.1	2935	97	1678	131	4841
		>-2	<-3	w/a	**3.3**	2.4 - 4.5	2935	97	828	90	3950
		>-3	<-3	w/a	**3**	2.2 - 4	3785	138	828	90	4841
		>-2	<-2 to <-3	h/a	**0.9**	0.4 - 1.9	1464	38	460	11	1973
		>-2	<-2	h/a	**1.3**	0.8 - 2.1	1464	38	1213	41	2756
		>-2	<-3	h/a	**1.5**	0.9 - 2.6	1464	38	753	30	2285
		>-3	<-3	h/a	**1.6**	1 - 2.5	1924	49	753	30	2756

Study	Measure	Control group	Malnourished group	Malnutrition Category	Unadjusted OR	95% CI	Control Number Survived	Control Number Died	Malnourished Number Survived	Malnourished Number Died	Total number
Demers, 2000 [29]	z-score SD	>-2	<-2	w/h	**3.2**	1.4 - 7.1	296	31	36	12	375
		>-2	<-2	h/a	**0.8**	0.4 - 1.6	224	33	112	13	382
Duke, 2001* [30]	% expected	>80%	60-80%	w/a	**2.5**	1.2 - 5.2	466	20	156	17	659
		>80%	<80%	w/a	**3.4**	1.8 - 6.5	466	20	180	26	692
		>80%	<60%	w/a	**8.7**	3.1 - 22.6	466	20	24	9	519
		>60%	<60%	w/a	**6.3**	2.4 - 15.2	622	37	24	9	692
Johnson, 2008 [31]	% expected	>80%	60-80% (no oedema)	w/a	**3.6**	1.2 - 12.9	136	5	127	17	285
		>80%	<80% (or oedema)	w/a	**5.2**	1.9 - 17.7	136	5	151	29	321
		>80%	<60% (or oedema)	w/a	**13.6**	3.9 - 52.7	136	5	24	12	177
		>60%	<60% (or oedema)	w/a	**6**	2.4 - 14.4	263	22	24	12	321
Kuti, 2013* [32]	z-score SD	>-2	<-2 to <-3	w/a	**0.9**	0.1 - 5.2	210	6	78	2	296
		>-2	<-2	w/a	**1.9**	0.6 - 6.7	210	6	164	9	389
		>-2	<-3	w/a	**2.8**	0.8 - 10.5	210	6	86	7	309
		>-3	<-3	w/a	**2.9**	0.9 - 9.5	288	8	86	7	389
		>-2	<-2 to <-3	w/h	**0.4**	0 - 3.7	221	6	84	1	312

Study	Measure	Control group	Malnourished group	Malnutrition Category	Unadjusted OR	95% CI	Control Number Survived	Control Number Died	Malnourished Number Survived	Malnourished Number Died	Total number
		>-2	<-2	w/h	**2.2**	0.7 - 7.5	221	6	153	9	389
		>-2	<-3	w/h	**4.3**	1.2 - 15.4	221	6	69	8	304
		>-3	<-3	w/h	**5.1**	1.5 - 16.9	305	7	69	8	389
		>-2	<-2 to <-3	h/a	**1.5**	0.3 - 6	276	10	56	3	345
		>-2	<-2	h/a	**1.4**	0.4 - 4.6	276	10	98	5	389
		>-2	<-3	h/a	**1.3**	0.1 - 6.5	276	10	42	2	330
		>-3	<-3	h/a	**1.2**	0.1 - 5.7	332	13	42	2	389
Lazzerini, 2016 [33]	z-score SD	>-2	<-2 to <-3	w/a	**2.4**	2.2 - 2.5	72509	3237	12509	1315	89570
		>-2	<-2	w/a	**3.1**	2.9 - 3.2	72509	3237	19854	2711	98311
		>-2	<-3	w/a	**4.3**	4 - 4.6	72509	3237	7345	1396	84487
		>-3	<-3	w/a	**3.5**	3.3 - 3.8	85018	4552	7345	1396	98311
Lupisan, 2007 [34]	z-score SD	>-2	<-2	w/a	**1.9**	0.8 - 4.1	863	17	355	13	1248
Man, 1998 [35]	z-score SD	>-2	<-2 to <-3	w/a	**1.6**	1 - 2.6	1225	56	472	35	1788
		>-2	<-2	w/a	**2.6**	1.8 - 3.7	1225	56	815	97	2193
		>-2	<-3	w/a	**4**	2.7 - 5.9	1225	56	343	62	1686
		>-3	<-3	w/a	**3.4**	2.3 - 4.8	1697	91	343	62	2193

Study	Measure	Control group	Malnourished group	Malnutrition Category	Unadjusted OR	95% CI	Control Number Survived	Control Number Died	Malnourished Number Survived	Malnourished Number Died	Total number
Naheed, 2009 [36]	z-score SD	>-3	<-3	w/a	**4.6**	2.9 - 7.3	3824	123	181	27	4155
Nantanda, 2008 [37]	z-score SD	>-3	<-3	w/a	**15.1**	4.9 - 50.8	111	6	22	18	157
Nathoo, 1993** [38]	% expected	>60%	<60%	w/a	**5.7**	3.3 - 9.7	556	66	49	33	704
Post, 1992 [39]	z-score SD	>-2	<-2 to <-3	w/a	**2**	1 - 4.1	91	54	22	26	193
		>-2	<-2	w/a	**3.5**	2 - 6.2	91	54	35	73	253
		>-2	<-3	w/a	**6.1**	2.9 - 13.3	91	54	13	47	205
		>-3	<-3	w/a	**5.1**	2.5 - 10.9	113	80	13	47	253
Ramachandran, 2012 [40]	z-score SD	>-2	<-2 to <-3	w/a	**1.3**	0.9 - 1.7	2005	126	1037	83	3251
		>-2	<-2	w/a	**1.8**	1.5 - 2.3	2005	126	1818	210	4159
		>-2	<-3	w/a	**2.6**	2 - 3.4	2005	126	781	127	3039
		>-3	<-3	w/a	**2.4**	1.9 - 3	3042	209	781	127	4159
Sigauque, 2009 [41]	z-score SD	>-3	<-3	w/h	**2.4**	1.3 - 4.4	495	95	43	20	653
Tupasi, 1988 [43]	% expected	>74%	60-74%	w/a	**3.6**	1.4 - 9.9	418	8	206	14	646
		>74%	<75%	w/a	**4.9**	2.1 - 12.7	418	8	277	26	729
		>74%	<60%	w/a	**8.8**	3.2 - 25.7	418	8	71	12	509

Study	Measure	Control group	Malnourished group	Malnutrition Category	Unadjusted OR	95% CI	Control Number Survived	Control Number Died	Malnourished Number Survived	Malnourished Number Died	Total number
		>60%	<60%	w/a	**4.8**	2.1 - 10.6	624	22	71	12	729
Tupasi, 1990 [42]	SD/ z-score	>-2	<-2 to <-3	w/a	**2**	1 - 4.1	161	17	123	26	327
		>-2	<-2	w/a	**2.4**	1.3 - 4.5	161	17	279	71	528
		>-2	<-3	w/a	**2.7**	1.5 - 5.3	161	17	156	45	379
		>-3	<-3	w/a	**1.9**	1.2 - 3.1	284	43	156	45	528
		>-2	<-2	h/a	**1.1**	0.7 - 1.8	252	48	181	37	518
		>-2	<-2 to <-3	h/a	**0.8**	0.4 - 1.5	252	48	93	14	407
		>-2	<-3	h/a	**1.4**	0.8 - 2.5	252	48	88	23	411
		>-3	<-3	h/a	**1.5**	0.8 - 2.5	345	62	88	23	518
Webb, 2012*^ [44]	z-score SD	>-2	<-2 to <-3 (no oedema)	w/a	**0.9**	0.2 - 3.1	322	12	119	4	457
		>-2	<-2 (or oedema)	w/a	**2.8**	1.3 - 6.3	322	12	212	22	568
		>-2	<-3 (or oedema)	w/a	**5.2**	2.3 - 12.2	322	12	93	18	445
		>-3	<-3 (or oedema)	w/a	**5.3**	2.5 - 11.6	441	16	93	18	568
		>-2	<-2 (or oedema)	w/h	**6.5**	2.4 - 19.3	369	7	121	15	512

Study	Measure	Control group	Malnourished group	Malnutrition Category	Unadjusted OR	95% CI	Control Number Survived	Control Number Died	Malnourished Number Survived	Malnourished Number Died	Total number
		>-2	<-2 to <-3	w/h	**3**	0.6 - 12.2	369	7	70	4	450
		>-2	<-3 (or oedema)	w/h	**11.4**	3.8 - 35.9	369	7	51	11	438
		>-3	<-3 (or oedema)	w/h	**8.6**	3.2 - 23	439	11	51	11	512
		>-2	<-2	h/a	**1.2**	0.4 - 3.1	320	13	169	8	510
		>-2	<-2 to <-3	h/a	**0.8**	0.1 - 3	320	13	93	3	429
		>-2	<-3	h/a	**1.6**	0.4 - 5	320	13	76	5	414
		>-3	<-3	h/a	**1.7**	0.5 - 5	413	16	76	5	510
Zabihullah, 2017 *^ [45]	z-score SD	>-2	<-2 to <-3	w/a	**2.4**	1.3 - 4.4	340	34	96	23	493
		>-2	<-2	w/a	**2.1**	1.2 - 3.5	340	34	199	41	614
		>-2	<-3	w/a	**1.7**	0.9 - 3.3	340	34	103	18	495
		>-3	<-3	w/a	**1.3**	0.7 - 2.4	436	57	103	18	614
		>-2	<-2 to <-3	w/h	**1**	0.1 - 4.7	340	13	51	2	406
		>-2	<-2	w/h	**1.2**	0.3 - 3.6	340	13	113	5	471
		>-2	<-3	w/h	**1.3**	0.2 - 4.8	340	13	62	3	418
		>-3	<-3	w/h	**1.3**	0.2 - 4.6	391	15	62	3	471
		>-2	<-2 to <-3	h/a	**12.2**	2.8 - 72.4	292	3	64	8	367

Study	Measure	Control group	Malnourished group	Malnutrition Category	Unadjusted OR	95% CI	Control Number Survived	Control Number Died	Malnourished Number Survived	Malnourished Number Died	Total number
		>-2	<-2	h/a	**9.5**	2.7 - 51.4	292	3	164	16	475
		>-2	<-3	h/a	**7.8**	1.8 - 46.2	292	3	100	8	403
		>-3	<-3	h/a	**2.6**	0.9 - 7.3	356	11	100	8	475

w/a – weight-for-age, w/h – weight-for height, h/a – height-for-age, SD – standard deviation, OR – odds ratio, CI – confidence intervals

* additional data provided by study authors

** original numbers of cases and controls imputed from sample size, relative risk and confidence intervals reported in study [11]

^ studies have missing weight-for-height and height-for-age measurements for several participants. Authors explain that this missing data is not random and that several children (more often children with pneumonia and malnutrition) were too sick for reliable admission height measurement. [49,50]

Supplementary Appendix 1 – Search Strategies

MEDLINE

1. pneumonia.mp. [mp=ti,ab,ot,nm,hw,px,rx,ui,tn,dm,mf,dv,kw]

2. exp pneumonia/

3. pneumon$.mp. or respiratory tract infection$.mp. or acute respiratory infection$.mp. or arti.mp. or acute respiratory tract infection$.mp. or acute lower respiratory tract infection$.mp. or acute lower respiratory infection$.mp. or alri.mp. or lri.mp. or lower respiratory infection$.mp.

4. exp respiratory tract infections/

5. exp bronchiolitis/ or exp bronchiolitis, viral/ or exp bronchiolitis obliterans/ or exp bronchitis/

6. bronchi*tis.mp.

7. 1 or 2 or 3 or 4 or 5 or 6

8. exp malnutrition/ or exp undernutrition/

9. malnutrition.mp. or kwashiorkor.mp. or marasmus.mp.

10. exp kwashiorkor/ or exp marasmus/ or exp protein-energy malnutrition/ or nutrition disorders/ or exp wasting disease/

11. undernutrition.mp. or malnourish$.mp. or undernourish$.mp. or underweight.mp. or stunt$.mp. or wast$.mp. or marasm$.mp. or thin$.mp.

12. 8 or 9 or 10 or 11

13. morbidity/ or incidence/ or *prevalence/* or mortality/ or "cause of death"/ or child mortality/ or exp infant mortality/

14. hospital admission.mp. or admission.mp. or hospital$.mp.

15. infant adj3 mortality.mp. or child adj3 mortality.mp.

16. *(burden adj3 disease).mp.*

17. (cause fatality or case fatality).mp. or fatal$.mp.

18. 13 or 14 or 15 or 16 or 17

19. 7 and 12 and 18

EMBASE

1. pneumonia.mp. [mp=ti,ab,ot,nm,hw,px,rx,ui,tn,dm,mf,dv,kw]

2. exp pneumonia/

3. pneumon$.mp. or respiratory tract infection$.mp. or acute respiratory infection$.mp. or arti.mp. or acute respiratory tract infection$.mp. or acute lower respiratory tract

infection$.mp. or acute lower respiratory infection$.mp. or alri.mp. or lri.mp. or lower respiratory infection$.mp.

4. exp respiratory tract infections/

5. exp bronchiolitis/ or exp bronchiolitis, viral/ or exp bronchiolitis obliterans/ or exp bronchitis/

6. bronchi*tis.mp.

7. 1 or 2 or 3 or 4 or 5 or 6

8. exp malnutrition/ or exp undernutrition/

9. malnutrition.mp. or kwashiorkor.mp. or marasmus.mp.

10. exp kwashiorkor/ or exp marasmus/ or exp protein-energy malnutrition/ or nutrition disorders/ or exp wasting disease/

11. undernutrition.mp. or malnourish$.mp. or undernourish$.mp. or underweight.mp. or stunt$.mp. or wast$.mp. or marasm$.mp. or thin$.mp.

12. 8 or 9 or 10 or 11

13. morbidity/ or incidence/ or *prevalence/* or mortality/ or "cause of death"/ or child mortality/ or exp infant mortality/ or exp hospital admission/

14. hospital admission.mp. or admission.mp. or hospital$.mp.

15. infant adj3 mortality.mp.

16. *(burden adj3 disease).mp.*

17. (cause fatality or case fatality).mp. or fatal$.mp.

18. 13 or 14 or 15 or 16 or 17

19. 7 and 12 and 18

GLOBAL HEALTH

1. exp malnutrition/ or exp protein energy malnutrition/ or exp undernutrition/ or exp nutritional state/ or exp nutritional disorders/ or exp wasting disease/

2. exp kwashiorkor/ or exp marasmus/

3.malnutrition.mp. or kwashiorkor.mp. or marasmus.mp. or undernutrition.mp. or malnourish$.mp. or undernourish$.mp. or underweight.mp. or stunt$.mp. or wast$.mp. or marsm$.mp. or thin$.mp.

4. 1 or 2 or 3

5. exp pneumonia/

6. pneumon$.mp. or respiratory tract infection$.mp. or acute respiratory infection$.mp. or arti.mp. or acute respiratory tract infection$.mp. or acute lower respiratory tract

infection$.mp. or acute lower respiratory infection$.mp. or alri.mp. or lri.mp. or lower respiratory infection$.mp.

7. exp bronchiolitis/

8. respiratory tract infection.mp. or bronchi*tis.mp.

9. (acute lower respiratory infection* or alri).mp. [mp=abstract, title, original title, broad terms, heading words]

10. 5 or 6 or 7 or 8 or 9

11 (cause fatality or case fatality).mp.

12 burden adj3 disease.mp. or infant adj3 mortality.mp.

13 hospital admission*.mp. or hospital admission.mp. or admission.mp. or hospital$.mp.

14. morbidity/ or incidence/ or disease incidence/ or "disease prevalence AND/OR seroprevalence" / or mortality/ or "cause of death"/ or child mortality/ or exp infant mortality/ or exp hospital admission/

15. 11 or 12 or 13 or 14
